# Antimicrobial Potential of *Streptomyces ansochromogenes* (PB_3_) Isolated From a Plant Native to the Amazon Against *Pseudomonas aeruginosa*

**DOI:** 10.3389/fmicb.2020.574693

**Published:** 2020-10-09

**Authors:** Erika Alves da Fonseca Amorim, Erima Joussiely Mendonça Castro, Silva Viviane da Souza, Mateus Silva Alves, Léo Ruben Lopes Dias, Maycon Henrique Franzoi Melo, Ilana Mirian Almeida da Silva, Paulo Cesar Mendes Villis, Maria Rosa Quaresma Bonfim, Angela Falcai, Maria Raimunda Chagas Silva, Valério Monteiro-Neto, Amanda Aliança, Luís Cláudio Nascimento da Silva, Rita de Cássia Mendonça de Miranda

**Affiliations:** ^1^Programa de Pós Graduação, Universidade CEUMA, São Luís, Brazil; ^2^Programa de Pós Graduação, Universidade Federal do Maranhão, São Luís, Brazil

**Keywords:** endophytes, biotechnological potential, actinomycetes, metabolites, endophytic, resistance

## Abstract

The objective of this study was to evaluate the antibacterial action of filamentous bacteria isolated from the *Byrsonima crassifolia* leaf. An endophytic bacterium has been identified by classical and molecular techniques as *Streptomyces ansochromogene*. Screening for antibacterial action against pathogens with medical relevance (*Klebsiella pneumoniae* ATCC 700603, *Pseudomonas aeruginosa* ATCC 15692, *Staphylococcus aureus* ATCC 6538, *Corynebacterium diphtheriae* ATCC 27012, *Mycobacterium abscessus*, *Cryptococcus gattii* ATCC 24065, and *Cryptococcus neoformans* ATCC 24067) demonstrated activity against the bacterium *P. aeruginosa* ATCC 0030 with inhibition diameter zones (IDZ) of 17.6 ± 0.25 mm in the preliminary screening in solid medium. After fermentation in liquid medium, an IDZ of 19.6 ± 0.46 mm and a minimum inhibitory concentration (MIC) of 0.5 mg/mL were detected. The antibiofilm action was observed with 100% inhibition of biofilm formation at a concentration of 0.250 mg/mL. When the infection curve was prepared, it was observed that the metabolite was effective in protecting the larvae of *Tenebrio molitor*. The metabolite does not show toxicity for eukaryotic cells. The leishmanicidal activity demonstrated that the metabolite presented a dose-dependent effect on the promastigotes forms of *Leishmania amazonensis* growth and the estimated IC_50_/72 h was 71.65 ± 7.4 μg/mL. Therefore, it can be concluded that the metabolite produced by the endophytic bacterium *Streptomyces* sp. is promising for future use as an alternative strategy against bacterial resistance.

## Introduction

Antimicrobial resistance is a dangerous and constantly increasing problem, leading to an increase in health care costs, decreased treatment options, and a higher incidence of mortality due to bacterial and fungal infections worldwide. Global initiatives are calling for urgent action not only to develop new antimicrobial therapies but also decrease the contribution of humans to increasing antimicrobial resistance (World Health Organization, 2015).

The excessive and inappropriate use of antibiotics continues to be a major factor in the expansion of antimicrobial resistance, and the search for new drugs has been an alternative to minimize the damage caused by this phenomenon.

Among the resistant microorganisms, a bacterium of most concern is *Pseudomonas aeruginosa.* According to Pang et al. (2019), *P. aeruginosa* is an opportunistic pathogen and a major cause of morbidity and mortality in patients with cystic fibrosis and immunocompromised individuals. The authors emphasize that the eradication of *P. aeruginosa* has become increasingly difficult due to its remarkable ability to resist antibiotics by intrinsic and acquired methods. The exchange of genetic material, which occurs naturally within or between species Gram-negative bacilli, is considered to be a factor responsible for the acquisition of resistance. Thus, the ability of *P. aeruginosa* to become resistant during antibiotic treatment is inherent to the species and is often inevitable (Lincopan and Trabulsi, 2008). In intensive care units (ICUs) in Brazilian hospitals, antibiotic resistance is very worrying.

National epidemiological studies carried out, aimed at hospitalized patients, evaluated 3,728 isolates, including Gram-positive and Gram-negative bacteria, obtained from 12 hospital centers in four states, and *P. aeruginosa* was responsible for 496 (13.3%) cases and the third most frequent pathogen, with 30.2% resistance to Integrated Pest Management (IPM) (Sader et al., 2001; Pang et al., 2019).

It is necessary to search for alternatives for treating diseases caused by these resistant microorganisms. Brazil is the country with the greatest biodiversity on the planet (Laihonen et al., 2004), and the Amazon ecosystem is one of the richest and most important because of its plant diversity that can house compounds with antimicrobial action.

The use of plants as a medicine is a practice adopted by traditional people; however, large-scale production of medicines is difficult. Therefore, the use of microorganisms that live inside the plant (endophytic) can be an alternative for the production of biologically active compounds through biotechnological techniques (Jiang et al., 2013; Hussain et al., 2015; Rajamani et al., 2018).

Among the microorganisms that produce bioactive compounds, those of the *Actinomycetales* family stand out, especially *Streptomyces* spp., which is known for its ability to produce substances with several antimicrobial, antimalarial, and antitumor actions (Supong et al., 2016; Elkhayat and Goda, 2017; Bunbamrung et al., 2020; Mahmood and Kataoka, 2020).

Considering these facts, this work aims to assess the antimicrobial potential of *Streptomyces ansochromogene*, isolated from *Byrsonima crassifolia* leaf, against pathogens of clinical interest.

## Materials and Methods

### Microorganisms

The microorganism used in this work is an endophytic bacterium previously isolated from *Byrsonima crassifolia* leaf, a plant native to the region of the Legal Amazon (Brazil). The bacterium is deposited in the culture bank of Ceuma University under the code PB_3_.

To test the antimicrobial potential of the isolated bacteria, the following microorganisms were used: *Klebsiella pneumoniae* ATCC 700603, *P. aeruginosa* ATCC 15692, *Staphylococcus aureus* ATCC 6538, *Corynebacterium diphtheriae* ATCC 27012, *Mycobacterium abscessus massiliense*Go01, *Cryptococcus gattii* ATCC 24065, and *Cryptococcus neoformans* ATCC 24067.

To assess the Leishmanicidal potential, *Leishmania amazonensis* (MHOM/BR/76/MA-76) was used.

### Identification of the Microorganism

#### Classical Identification

For classic identification of the isolate, the microculture technique was performed. Previously isolated and purified microorganism was inoculated in a Petri dish containing the BDA medium that favors radial growth. A coverslip was partially inserted into the medium to facilitate the growth of hyphae on its surface. This was incubated in an oven at 28°C for 5 days (Shirling and Gottlieb, 1966; Matsuura, 2004). Structures such as conidiospores, hyphae, spore chains, and conidia were stained with cotton blue and observed under an optical microscope with 100x magnification lenses. Genera level identification was possible by observation of macroscopic characteristics and microscopic morphological structures. For the morphological description of the genera, the criteria adopted by Rapper and Fennell, 1965, Pitt, 1979, Pitt and Samson, 1990 and Klich and Pitt, 1988.

#### Molecular Identification: DNA Extraction, Amplification of the 16sR Region, and Sequencing

Molecular identification was used to confirm the bacterial species previously identified by classical techniques. Thus, the bacteria were grown in Petri dishes for five days and incubated in PBS for 2 h at 27°C. After centrifugation at 7500 rpm for 10 min, supernatant was discarded and pellet was suspended in 180 μL of lysozyme (200 μg/mL), incubated for 30 min at 37°C. To this solution, 20 μL of proteinase K and another 200 μL of Buffer AL buffer solution (QIAmp) were added. The solution was homogenized and incubated for 30 min at 56°C, then for another 15 min at 95°C. The material was centrifuged and 200 μL of absolute ethanol was added and centrifuged again. The samples were transferred to the Purification Kit (QIAmp DNA mini kit) and centrifuged for 1 min at 8000 rpm. The filtrate was discarded, and 500 μL of AW2 buffer solution (QIAmp) was added and centrifuged for 3 min at 1400 rpm. The filtrate was discarded again and centrifuged for 1 min at 1400 rpm, and then 50 μL of Buffer AE buffer solution (QIAmp) was added and incubated for 1 min and centrifuged again for 1 min at 8000 rpm. This last step was repeated (1X). A small amount of the target DNA was added to a PCR buffer solution (10 mM Tris-HCl [pH 8.0], 50 mM KCl) containing DNA polymerase, universal primers P27F (5′-AGAGTTTGATCCTGGCTCA-3′), and P1492R (5′-GGTTACCTTGTTACGACTT-3′) (Lee et al., 2007). In PCR tubes, 0.25 mL of the mixture was placed, which was subjected to approximately 40 replication cycles *in vitro* (Thermociclador) for 1 min at 95°C, 45 s at 57°C, and 30 s at 72°C. DNA was quantified using NanoDrop^®^ ND-1000 UV-Vis. The samples were applied to an agarose gel immersed in a TBE buffer solution and electrophoresis was performed for 40 min. The gel was stained with UniSafedye (0.1 μL/mL) for 40 min, protected from light and read on an ultraviolet light transilluminator. The PCR product was sequenced and analyzed using MegaSoftware^®^ and Blast (NCBI). The sequence of the 16S rRNA gene obtained by PCR was analyzed and manually aligned with strains of the genus found in the GenBank/NCBI database with Clustral-X. The phylogenetic tree was built using MEGA version 7.0. The sequence was submitted to GENBANK/PUBMED and deposited under code MT634691.

### Preliminary Antimicrobial Screening

#### Solid Media Assay

Antimicrobial activity testing was performed on solid medium through the diffusion of the bioactive compound in agar using the method described by Ichikawa et al. (1971). This method is also known as the Gelose Block Method using the isolated endophytic microorganism. After ten days of incubation at 28°C, circular agar blocks of 6 mm in diameter were removed from the plates with colonies grown by clogging and transferred to the plates containing MH, previously seeded with the standard microorganisms at 5 × 10^5^ UFC/mL. This test was performed in triplicates. After 24 h at a temperature of 37°C, the diameters (mm) of the inhibition halos of each block were measured, analyzing the longest distance between 2 rectilinear points that cross the block of glucose in half. A Matsuura scale (2004) was used to classify the results, so that the arithmetic mean and standard deviation of the results were obtained. The results were expressed as mean inhibition diameter zones (IDZ) in millimeters (mm).

### Antimicrobial Activity

#### Submerged Fermentation

To obtain the active metabolite produced by the isolated microorganism, submerged fermentation was carried out in Erlenmeyer flasks (250 mL) containing 50 mL of useful volume of potato dextrose (PD) medium. The flasks were incubated in a rotary incubator at 30°C for 5 days. After this period, the sample was filtered to assess biological activities.

##### Secondary metabolites extraction

To obtain the metabolite(s) of interest, liquid–liquid extraction was performed according to the methodology described by Trisuwan et al. (2008). Therefore, after the incubation period, the fermented culture was filtered through a vacuum filter, and the free extract of microbial cells was separated. The fermented filtrated was subjected to centrifugation and filtered again in a 22 μm filter, ensuring that the extract did not contain microbial cells.

To extract the compounds of interest, 25 mL of the filtrate and 25 mL of ethyl acetate were added to a separating funnel, shaken vigorously for 10 min, and placed at rest for 30 min. After the mentioned time, the organic phase containing the analytes of interest was collected. The solvent was then evaporated on a rotary evaporator, and the product yield was determined.

For the evaluation of biological activities, the extract was resuspended in dimethylsulfoxide (DMSO) to reach a known concentration of 1 mg/mL.

##### Agar diffusion assay

The liquid medium assay was performed using the plate diffusion test (Bauer et al., 1966) to determine whether the microorganisms selected in the previous assay secrete metabolites to the external environment. The plate diffusion test was established as a standard by the Clinical and Laboratory Standards Institute (CLSI) and consists of the application of 10 μL of the metabolite produced in 6 mm diameter wells, made in petri dishes with 20 ml of Mueller Hinton agar medium, seeded with gram-negative bacteria, incubated at 37°C for up to 72 h. After this period, IDZs were measured with the aid of a caliper. In the 6 mm wells, 10 μL of DMSO and 10 μL of chloramphenicol (30 μg) were used as negative and positive controls, respectively.

#### Determination of Minimum Inhibitory Concentration

For the determination of the minimum concentration capable of inhibiting bacterial growth, the microdilution test was performed, using a 96-well 6-mm multi-well plate, the metabolite was diluted in dimethyl sulfoxide (DMSO). The calculation to determine the concentration followed the CLSI protocol, which recommends 1000 μg/mL.

The technique described by Zgoda and Porter (2001) was adapted. Mueller Hinton broth (190 μL) and the metabolite (10 μL) diluted in DMSO at an initial concentration of 1000 μg/mL were dispensed in the first row of wells. In the other wells, 100 μL of medium Muller Hinton broth was added. A serial dilution was then performed in the nine consecutive wells, removing 100 μL from the well of the highest concentration, resulting in dilutions from 1000 μg/mL to 0.0625 μg/mL. In the penultimate well, the inoculum and extract were not added to have a negative control. In the last well, medium and inoculum were added as positive control. Microbial growth was determined by bacterial colony growth. The concentration of the bacterial suspension was determined according to the McFarland scale 5 × 10^5^ CFU/mL. The plate was incubated at 37°C for 24–48 h.

### Evaluation of Antibiofilm Activity

The inhibitory capacity of the metabolite in biofilm formation was assessed according to the method recommended by Stepanoviæ et al. (2007). The method consists of adding 100 μL of Broth Muller Hinton medium to all 96 wells of a multi-well plate. The first and last rows are the negative and positive controls, respectively. In the second row, another 50 μL of culture medium was added, totaling 150 μL of medium. Then, 50 μL of the metabolite in sub-MICs was added to this second row, totaling a volume of 200 μL in the second row. Serial dilution was performed up to the penultimate row before the positive control. After diluting 100 μL of the inoculum on a 0.3 × 10^8^ scale, all wells were added, except for the negative control. The plate was incubated at 37°C for 24 h. After this period, the contents of the plate were removed, and the wells were washed with PBS buffer or saline solution. To fix the biofilm, 200 μL of methanol was placed in the wells for 15 min at room temperature. This was washed with PBS or saline again. The wells were stained with crystal violet and incubated for 15 min at 37°C. They were then washed in running water to remove excess dye. After drying, 250 μL of ethanol was used for suspension in the wells, and ethanol was transferred to a new plate that was taken to read the optical density in a 550 nm microplate reader. To determine the cell viability of bacterial biomass, the test was repeated and the crystal violet was replaced by resazurin indicator.

### Action of Metabolite on Biofilm

Eradicating the action of preformed biofilm was assessed using the method of Klein et al. (2015), with modifications. It was divided into two stages: 1. For the previous formation of the biofilm and 2. Evaluating the eradication of the formed biofilm. The first step consisted of the formation of the biofilm in a 96-well plate, where 100 μL of Muller Hinton broth culture medium was added to all wells, and the first and last rows were the negative and positive controls, respectively. Then, 100 μL of the standardized microbial inoculum was added to 0.3 × 10^8^ cells, except in the first row, which was the negative control. This plate was incubated at 37°C for 24 h. Later, the second stage began, where the contents of the plate were removed, and the wells were washed with PBS buffer or saline solution. Then, 100 μL of Muller Hinton broth medium was added to the washed wells. In the second row of the wells, the metabolite was placed in supra-MIC (4 × MIC), followed by serial dilution. This plate was incubated again for 24 h at 37°C.

After this step, the contents of the wells were gently removed and washed with PBS buffer. Then, 200 μL of methanol was placed for 15 min at room temperature. The wells were again washed with PBS buffer, stained with crystal violet, and incubated at 37°C for 15 min. After that, the plate was washed under running water, and after drying, the wells received 250 μL of ethanol. This suspension was transferred to a new plate and was taken to read optical density in an Elisa reader at 550nm.

### Infection and Survival Curve in an Alternative Model

To standardize the inoculum concentration scale in its pathogenic potential of *P. aeruginosa*, 5 standardized solutions were prepared of 0.1, 0.2, 0.3, 0.3 and 0.5 × 10^8^ cells using Newbauer Chamber. Each concentration was inoculated into 10 larvae of *T. molitor* to observe mortality levels. The smallest scale that obtained the ability to kill *T. molitor*, in less days of observation, was the inoculum scale used for infection and survival curves (Figure 4A). Having the inoculum scale defined previously, four groups composed of 10 *T. molitor* each were established. Of these, three groups were infected with the pathogen, after 2 h of infection, one group was treated with 10 μL of the test extract, another group received no treatment, and the fourth group was not infected, for quality control of *T. molitor*. They were observed for 10 days, where the survival time was observed.

### Leishmanicidal Activity Against Promastigotes

To determine the IC50 (a concentration that inhibits 50% of parasite growth) value, promastigote forms of *Leishmania amazonensis* (MHOM/BR/76/MA-76) were incubated in the presence of increasing concentrations of the secondary metabolite (7.8–500 μg/mL). The cells were diluted at a concentration of 1 × 10^6^ parasites/mL. As a control, cells incubated with Schneider’s medium were used. After 72 h of incubation the surviving parasites were counted in a Neubauer chamber (INCYTO CChip DHC-N01, Cheonan-Si, South Korea). The IC50 value was determined by linear regression analysis using SPSS 8.0 software (IBM Co., New York, United States) for Windows. Each experiment was performed in biological duplicate and technical triplicate.

### Cytotoxicity Assay

The MTT method is based on the determination of the ability of living cells to reduce 3-(4,5 Dimethyl Thiazol-2-IL)-2,5-Diphenyl Tetrazolium (MTT) bromide forming insoluble crystals of formazan violet. After treatment, the culture medium was removed and a 10% solution of MTT (5mg/mL) in PBS was added to each well. Then, the cultures were incubated at 37°C for 3 hours, protected from light. For solubilization of formazan crystals, 100 μL of dimethylsulfoxide (DMSO) was added to each well and the presence of violet formazan crystals was observed. The spectrophotometric reading of the absorbance was performed in an ELISA plate reader (Bio-Rad Microplate Reader Benchmark, Inc., United States) at a wavelength of 550 nm. The percentage of dead cells was calculated in relation to the positive toxicity control.

## Results

### Microorganism Identification

#### Classical Identification

**FIGURE 1:**
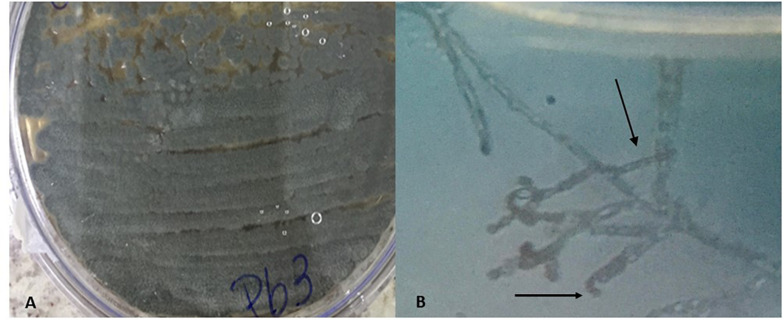
Macroscopic characteristic with aerial mycelium of grayish color showing the production of pigment **(A)** and microscopic with spiral-shaped sporophores characteristic of bacteria of the genus *Streptomyces* **(B)**.

The classical identification of microorganism was carried out through macro and microscopic observation of the colonies. Features such as colony color, pigment production in the culture medium, and microscopic structures were considered. Macroscopic observation of the colony in a Petri dish showed a grayish colony, with predominant aerial mycelium and dark pigment release (Figure 1A). On microscopic observation, it was possible to identify the gray spore chain structures, branched mycelia similar to filamentous fungus hyphae, characteristics compatible with actinobacteria of the genus *Streptomyces* (Figure 1B) (Olmos et al., 2013; Raju et al., 2014). Spores similar to those of fungi, known as astropores and spiral-shaped sporophores, were also observed (Walkman & Henrici, 1943; Ensign, 1978).

#### Molecular Identification by PCR

**FIGURE 2:**
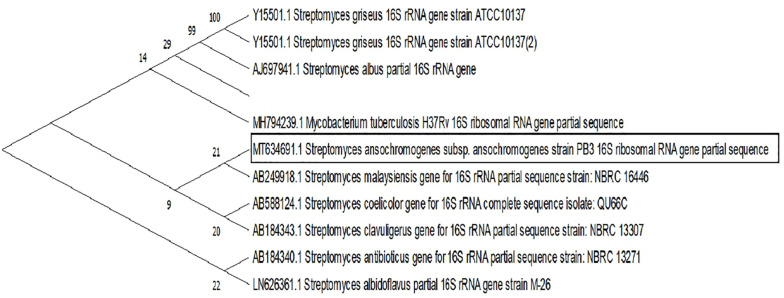
Phylogenetic tree likelihood, based on the sequences of the 16S rRNA gene relating eight strains of bacteria of the genus *Streptomyces*. Branch nodes have an initialization value based on 1000 resampled datasets.

Molecular identification demonstrated a 97.11% genetic similarity with *Streptomyces ansochromogene*. Then, macroscopic identification of the strain genus was confirmed. The evolutionary analysis was performed in MEGA X. A total of 1510 positions were present in the final dataset, involving 7 nucleotide sequences, and the codon positions included were 1st + 2nd + 3rd + uncoded. Evolutionary history took place using the maximum likelihood method and the Tamura and Nei (1993). The tree for heuristic research was automatically generated by Join-Join, BioNJ, and distance matrix in estimated pairs using the maximum likelihood approach, selecting the topology most likely to log (2471.39) (Figure 2).

### Screening of Antimicrobial Potential of Isolated Bacteria

The antimicrobial potential of the *S. ansochromogene* was evaluated using a solid test against seven microorganisms of clinical interest. *S. ansochromogene* was shown to be active for all microorganisms tested, except for *Cryptococcus neoformans* ATCC 24067 (Table 1). The higher activities were observed for *M. abscessus, C. diphtheriae* ATCC 27012, and *K. pneumoniae* ATCC 700603 with IDZs of 39.3 ± 0.11 mm, 38.3 ± 0.28 mm, and 30 ± 0 mm, respectively. In this test, the bacterium was also active against the pathogenic yeast *C. gattii* 24065 and the bacteria *P. aeruginosa* ATCC 15692 and *S. aureus* ATCC 6538 with IDZs of 26 ± 0.1, 14.3 ± 0.11, and 17.6 ± 0.25, respectively.

**TABLE 1:** Diameters of inhibitions zones formed by *Streptomyces ansochromogene* against the pathogens tested in medium solid assays.

**Microorganisms pathogens**	**PB3**
*Klebsiella pneumoniae* (ATCC 700603)	30 ± 0
*Pseudomonas aeruginosa* (ATCC 15692)	17.6 ± 0.25
*Staphylococcus aureus* (ATCC 6538)	14.3 ± 0.11
*Corynebacterium diphtheriae* (27012)	38.3 ± 0.28
*Mycobacterium abscessus (IC)*	39.3 ± 0.11
*Cryptococcus neoformans* (24067)	0
*Cryptococcus gattii* (24065)	26 ± 0.1

### Agar Diffusion Assay

To investigate whether *S. ansochromogene* PB3 secreted the active metabolite (s), a liquid test was performed followed by liquid-liquid extraction with ethyl acetate. The crude extract was obtained with a yield of 0.16 g/L and tested against *K. pneumoniae* (ATCC 700603); *P. aeruginosa* (ATCC 15692); *C. diphtheriae* (ATCC 27012); *M. abscessus* and *C. gattii* (ATCC 24065). Showing inhibitory activity against *P. aeruginosa* ATCC 15692 (IDZ 19.6 ± 0.47 mm) (Table 2).

**TABLE 2:** Diameters of inhibitions zones formed by *Streptomyces ansochromogene* against the pathogens tested in medium liquid assays.

**Microorganisms pathogens**	**PB3**
*Klebsiella pneumoniae* (ATCC 700603)	0
*Pseudomonas aeruginosa* (ATCC 15692)	19.6 ± 0.47
*Staphylococcus aureus* (ATCC 6538)	0
*Corynebacterium diphtheriae* (ATCC 27012)	0
*Mycobacterium abscessus (IC)*	0
*Cryptococcus gattii* (ATCC 24065)	0

### Determination of Minimum Inhibitory Concentration

To evaluate the lowest concentration that inhibited the growth of *P. aeruginosa* (ATCC 15692) determined in the previous test, the minimum inhibitory concentration (MIC) was evaluated according to the CLSI protocol. The end point of the MIC was considered to be its lowest concentration, where there was no color change. All tests were performed in triplicates. The minimum inhibitory concentration was 0.5 mg/mL for the extracted metabolite.

### Anti-biofilm Activity

**FIGURE 3:**
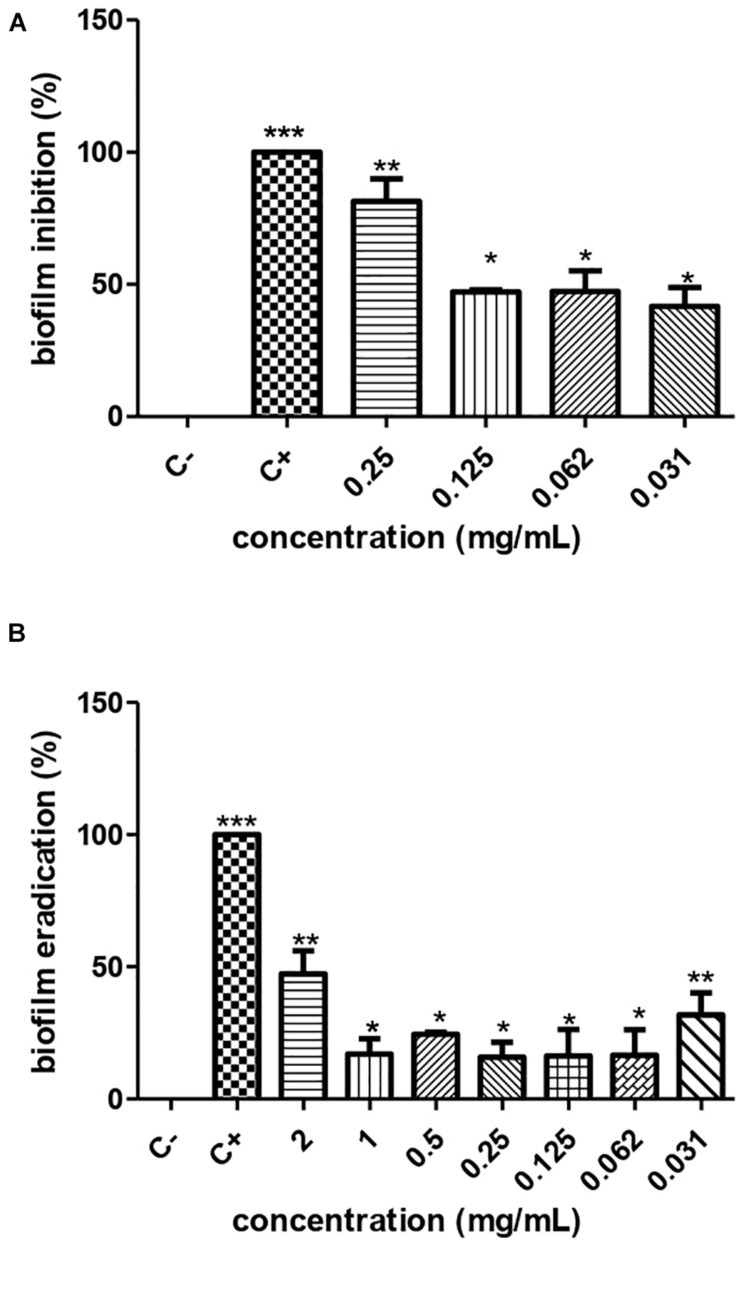
Inhibition **(A)** and eradication **(B)** of the biofilm of *P. aeruginosa* ATCC 0030 by different concentrations of the metabolite produced by *Streptomyces ansochromogenes*. All experiments were performed in triplicate and for statistical analysis, *p* < 0.05 was considered.

According to the crystal violet technique, the metabolite produced by *S. ansochromogene* PB3 inhibited the biofilm formed by *P. aeruginosa*, at sub-MICs of 0.25 mg/mL, 0.125 mg/mL, 0.0625 mg/mL, and 0.0312 mg/mL, eradicating 82.54%, 46.53%, 33.44%, and 27.98%, respectively. This presented antimicrobial activity on *P. aeruginosa* biomass (ATCC 15692). The cellular infeasibility of biomass in the first inhibitory concentrations was confirmed by a test with resazurin (Figure 3A).

### Action of Metabolite on Biofilm

The metabolite produced by *S. ansochromogene* PB3 reduced 50% of the biofilm formed, at a hyperinhibitory concentration of 2 mg/ml (Figure 3B). The resazurin test confirmed the cellular infeasibility of the biofilm bacterial biomass at this concentration.

### Infection and Survival Curves in an Alternative Model

**FIGURE 4:**
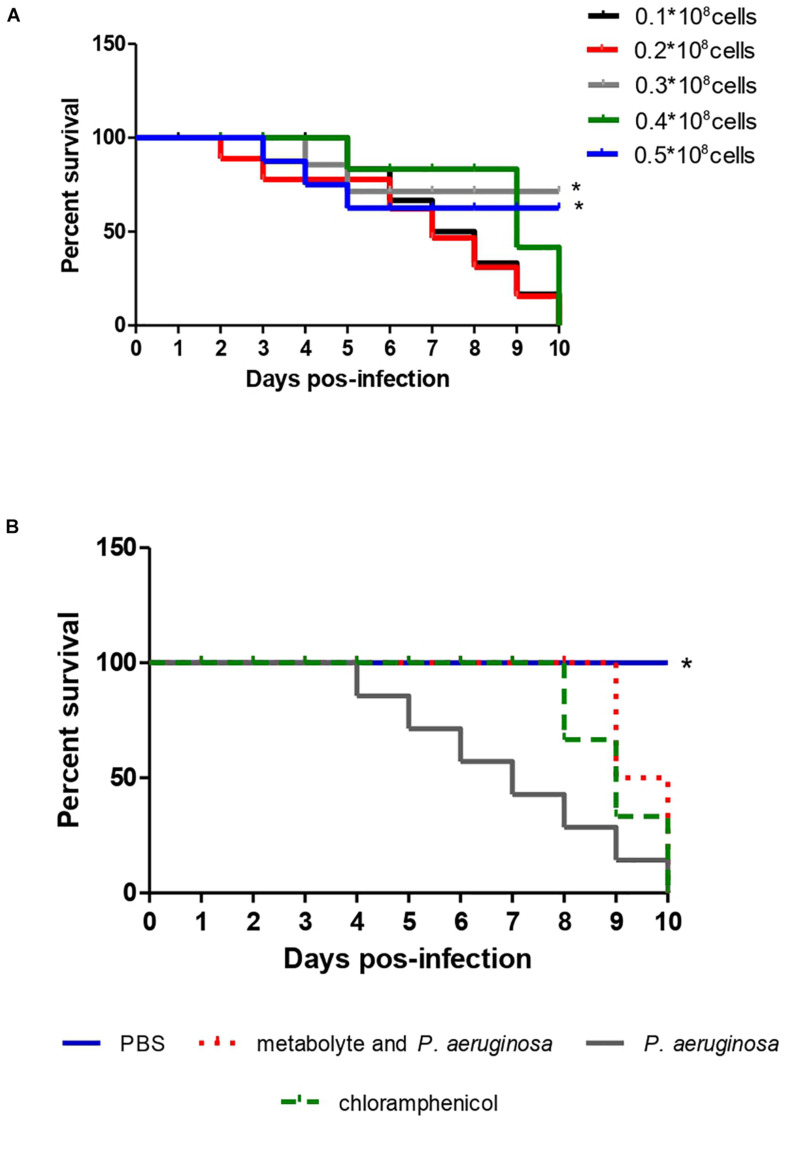
Survival curve of 10 *T. molitor* larvae infected with different concentrations of *P. aeruginosa* cells ATCC 0030 for standardization of the inoculum **(A)** and for treatment with metabolite produced by *Streptomyces ansochromogenes* **(B)**. All experiments were carried out in triplicate for statistical analysis, *p* < 0.05 was considered.

The metabolite produced by the strain of *S. ansochromogene* at a concentration of 1 mg/L showed the ability to increase the life span of *T. molitor* infected with the pathogen *P. aeruginosa*. The larvae infected with a standard solution of 0.3 × 10^8^ of *P. aeruginosa* were all dead on the eighth day but those infected with the bacterium and inoculated with the metabolite survived until the eighth day; two larvae died on the ninth and tenth days, a behavior similar to the larvae infected with *P. aeruginosa* and treated with chloramphenicol (Figure 4B). When the statistical analysis was performed, a significant difference was observed between the treatment of the larva with the metabolite in relation to the larva infected with *P. aeruginosa* (*p* = 0.0082); however, there was no difference when comparing the treatment of the larva with the metabolite and chloramphenicol.

### Leishmanicidal Activity Against Promastigotes

**FIGURE 5:**
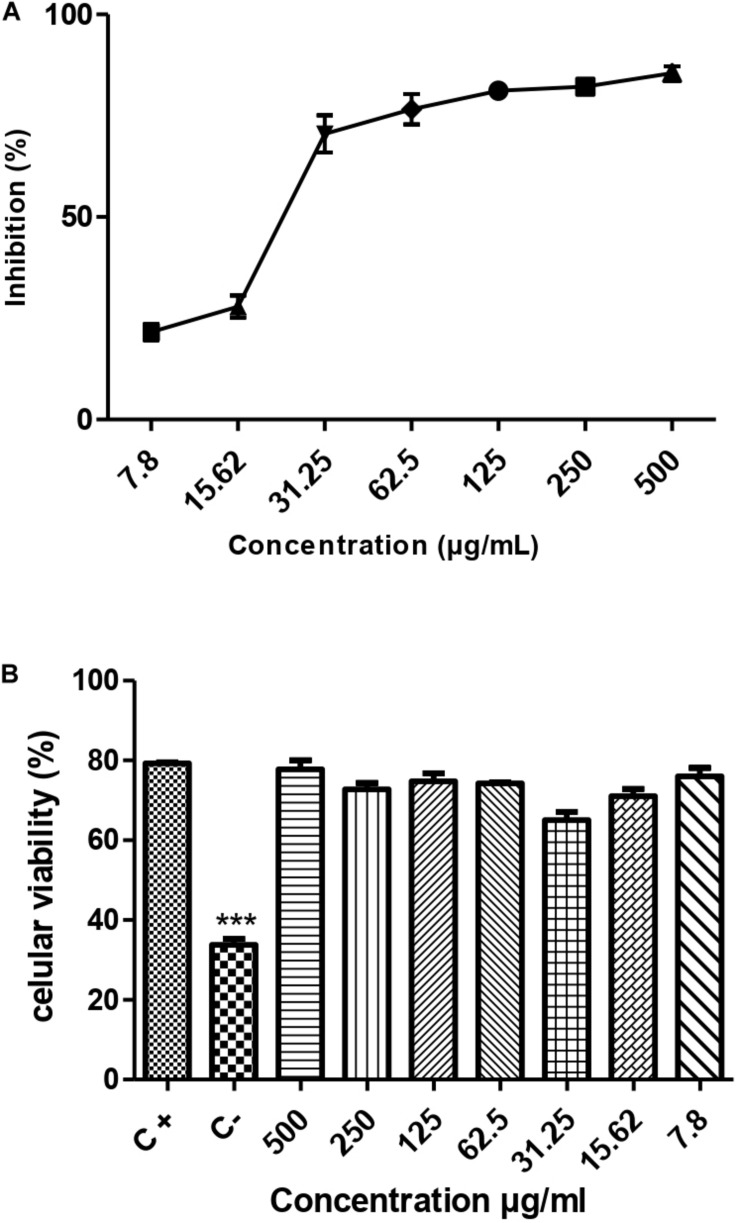
Leishmanicidal activity **(A)** and cytotoxicity **(B)** of the metabolite produced by the endophytic bacteria *Streptomyces ansochromogenes* (PB3). All experiments were carried out in triplicate for statistical analysis, *p* < 0.05 was considered.

The results demonstrated that the metabolite presented a dose-dependent effect on the promastigotes forms of *L. amazonensis* (MHOM/BR/76/MA-76) growth; it is possible to observe that in the five highest concentrations the metabolite was able to reach more than 50% growth inhibition (Figure 5A). The estimated IC_50_/72 h was 71.65 ± 7.4 μg/mL.

### Cytotoxicity Assay

The toxicity test was carried out to assess the potential of the metabolite to cause damage to cells of eukaryotic organisms. The result of the cytotoxicity test showed that the metabolite produced by the bacterium *S. ansochromogene* is not toxic to the cell at any concentration tested (Figure 5B).

## Discussion

The bacterium used in this work was isolated from a leaf of *Byrsonima crassifolia* and was shown to be promising against *P. aeruginosa*. Endophytic microorganisms have been increasingly studied owing to their potential as excellent agents with diverse biological activities (Gos et al., 2017; Pinheiro et al., 2017; Rajamani et al., 2018; Singh et al., 2018). To identify the isolated bacterium, classical and molecular identification was performed, obtaining the bacterium *S. ansochromogene*. Molecular identification was used as the main tool for identifying these bacteria because in many cases, they can be confused with fungi. Van Nguyen et al. (2019) reported the genome sequence of a endophytic *Streptomyces* sp. that produced a bioactive compound of clinical interest. Bacteria of the genus *Streptomyces* are known to produce bioactive compounds with diverse biological activities. Its morphology and high metabolic potential favor the permanence of the bacteria inside the plant where there is a symbiotic relationship between the plant and the microorganism. Several authors have reported the isolation of bacteria of this genus associated with plants used by humans for various purposes. Lei et al. (2016) isolated a *Streptomyces* sp. from a medicinal plant (*Polygonum cuspidatum*) and observed its antimicrobial activity against Gram-negative bacteria *Escherichia coli*. Al-Ansari et al. (2019) reported the isolation of ten actinomycetes from the soil, of which five demonstrated activity against *Enterobacter aerogenes* and *Proteus mirabilis*. Kalyani et al. (2019) isolated 143 actinomycetes, including a *Streptomyces* sp. NLKPB-45 showed activity against Gram-positive bacteria, *S. aureus*, and Gram-negative *Escherichia coli* and *P. aeruginosa*.

In this study, the bacterium isolated from the leaf of *Byrsonima crassifolia* was identified as *S. ansochromogene* and showed activity against several pathogens of clinical interest, including the Gram-negative bacterium *P. aeruginosa*. This makes the study of the metabolite produced by the bacteria promising.

The prospecting of compounds with activity against *P*. *aeruginosa* has been increasingly intensified due to the phenomenon of resistance to antimicrobials that these bacteria have demonstrated. Neves et al. (2011) reported the phenomenon of resistance to antimicrobials by this bacterium as an endemic, affecting an important portion and with strains resistant to the main antibiotics for clinical use. Although the search for active compounds is reported in the literature, there are not many studies showing activity of bacteria of the genus *Streptomyces* sp. against *P. aeruginosa*. Kalyani et al. (2019) reported the activity of *Streptomyces* sp. NLKPB-45 against Gram-negative bacteria *Escherichia coli* and *P. aeruginosa*. Ashitha et al. (2019) isolated endophytic bacteria from the Chinese medicinal plant *Artemisia nilagirica* (Clarke) Pamp. Among the isolates, the bacterium *Burkholderia* sp. showed activity against several pathogens of clinical interest, including the bacterium *P. aeruginosa* MTCC 2453.

The concentration at which the metabolite shows activity is extremely important to assess its viability. In this work, extraction of the active fraction of the metabolite was performed using ethyl acetate solvent to determine the lowest concentration that showed activity (MIC). Lei et al. (2016) reported the activity of the metabolite acetate fraction produced by *Streptomyces* sp. A09916 against Gram-negative bacteria *Salmonella* sp. S11A235 at a concentration of 0.125 mg/L. Kumar et al. (2020) obtained the active fraction of the metabolite produced by *Streptomyces olivaceus* LEP 7 with a Minimum Inhibitory Concentration of 50 μg/mL against the bacterium *P. aeruginosa*.

One of the main problems that make infections by the *P. aeruginosa* bacteria even more difficult to treat is that, in addition to the resistance phenomenon, these bacteria also have the ability to form biofilms due to the high capacity to carry out the quorum sense due to the glycidic nature of its cell wall (Satpathy et al., 2016). Given this characteristic, it is important to investigate the ability of the metabolite to prevent formation or eradicate the biofilm. In this study, the acetate fraction extracted from the metabolite of the endophytic bacteria *S. ansochromogene* showed the ability to inhibit and eradicate biofilms formed by *P. aeruginosa* at subinhibitory concentrations. The two phenomena (inhibition and eradication of biofilm) are related to the tested concentration of the metabolite, which can be explained by the selectivity of the membrane, which allows the metabolite to penetrate the cell at lower concentrations, making it more efficient. Antibiofilm activity has been investigated and reported by several authors. Æiriæ et al. (2019) reported the importance of the search for metabolites that inhibit the phenomenon of quorum sense, the first step in the formation of biofilms. The authors emphasize that many plants already have this known activity; however, the search for metabolites produced by microorganisms with this activity should be intensified. Younis et al. (2016) reported the metabolite antibiofilm activity produced by a species of *Streptomyces* sp. isolated from a marine environment. The authors emphasized the ability of the metabolite acetate fraction to inhibit the phenomenon of quorum sense, thus preventing the formation of biofilm. Pattnaik et al. (2018) reported the antibiofilm activity of the extract of the metabolite produced by the fungus *Diaporthe phaseolorum* SSP12 against a biofilm formed by *P. aeruginosa* PAO1. The authors also emphasized the ability of the extract to inhibit 79% ± 3.5% quorum sensing formation in concentrations of 0.750 μg/mL, which is commonly performed by strains of these bacteria. The use of compounds with anti-biofilm activity has been reported as a possible alternative therapy for treating various infections as a solution to the phenomenon of microbial resistance (Santhakumari and Ravi, 2019).

Biofilm formation is a strategy used by *P. aeruginosa* to cause infections, so it is important to test the metabolite activity *in vivo*. In this study, the capacity of the *S. ansochromogene* metabolite was tested in an alternative model using *T. molitor* larvae. In this evaluation, it was observed that the metabolite at the concentration (0.5 mg/mL) established in the MIC had the capacity to inhibit the infection caused by *P. aeruginosa* bacteria in *T. molitor*. The larvae treated with the metabolite showed greater survival than those treated with the antibiotic chloramphenicol, emphasizing the protective effect of the metabolite. Because the use of animal models is decreasing for ethical reasons, alternative models have been reported in the literature as an excellent way to evaluate the effectiveness of compounds in living beings. Silva et al. (2019) assessed the ability of *S. aureus* to infect *Galleria mellonella* larvae. The authors observed the pathogen’s ability to infect and form biofilms inside the animal.

Leishmaniasis is a treatable and curable disease, but drugs that used in treatment have several limitations because of serious side effect (World Health Organization (WHO), 2018; Araújo et al., 2019). The results showed that the secondary metabolite inhibited the growth of promastigote forms, a fact observed by Santiago et al. (2012) that isolated 564 endophytic fungi from plants of the Antarctic, these 19 sources leishmanicidal activity against a promastigote form of *L. amazonensis*. This activity is reinforced by the cytotoxicity test that shows that the metabolite is not toxic to the cell at any concentration tested, suggesting its specificity for the protozoan.

## Conclusion

The endophytic bacterium *S. ansochromogene* has anti-microbial potential because it produces a metabolite that can be used for its antimicrobial and antibiofilm action against the bacterium *P. aeruginosa* and against *L. amazonensis* in promastigote form. In view of these results, studies will be carried out with the objective of identifying the compound and expanding its spectrum of action, evaluating whether it has activity against other protozoa and tumors.

## Data Availability Statement

The datasets presented in this study can be found in online repositories. The names of the repository/repositories and accession number(s) can be found below: https://www.ncbi.nlm.nih.gov/genbank/, MT634691.

## Author Contributions

This manuscript has as main objective to show the action of one or more compounds produced by a microorganism called *Streptomyces ansochromogenes* isolated from a plant in face of a common actia in contaminated wounds called *Pseudomonas aeruginosa*. This bacterium has the ability to form biofilms to be able to colonize contaminated wounds when an infection process begins, so it is important that compounds manage to prevent this mechanism. All authors contributed to the article and approved the submitted version.

## Conflict of Interest

The authors declare that the research was conducted in the absence of any commercial or financial relationships that could be construed as a potential conflict of interest.
